# Prevalence of *Xanthomonas euvesicatoria* (formally *X. perforans*) associated with bacterial spot severity in *Capsicum annuum* crops in South Central Chihuahua, Mexico

**DOI:** 10.7717/peerj.10913

**Published:** 2021-02-15

**Authors:** Jared Hernández-Huerta, Patricia Tamez-Guerra, Ricardo Gomez-Flores, Ma. Carmen E. Delgado-Gardea, Margarita S. García-Madrid, Loreto Robles-Hernández, Rocio Infante-Ramirez

**Affiliations:** 1 Facultad de Ciencias Agrotecnológicas, Universidad Autónoma de Chihuahua, Campus 1, Chihuahua, Chihuahua, México; 2 Facultad de Ciencias Biológicas, Universidad Autónoma de Nuevo León, San Nicolás de los Garza, Nuevo León, México; 3 Facultad de Ciencias Químicas, Universidad Autónoma de Chihuahua, Circuito Nuevo Campus, Chihuahua, Chihuahua, México

**Keywords:** *Xanthomonas*, Bacteriosis severity, Jalapeño pepper, Antibacterial agents, Copper resistance

## Abstract

**Background:**

*Xanthomonas* spp. causes bacterial spot disease, which reduces quality and yield of pepper crops in Mexico. Identification of phytopathogen species is necessary to implement more effective control strategies.

**Objective:**

The aim of this study was to isolate and identify infecting *Xanthomonas* species in South Central Chihuahua pepper-producing areas.

**Methods:**

Diseased plants were collected from 30 cultivation lots and bacteria were isolated from damaged tissues. Potential causative agents were isolated, identified, and characterized by biochemical and molecular analysis. Pathogenicity tests from each isolate were then performed on 30-d-old pepper seedlings, exposing five leaves to 10 µL of 1 × 10^8^ CFU/mL bacterial suspensions of each isolate, using sterile distilled water as a control. Disease severity was determined after 10 d by calculating leaf damage percentage. Furthermore, we evaluated the susceptibility of the highest bacterial spot severity-causing isolates (13 isolates) to copper sulphate (CuS), copper gluconate (CuG), copper oxychloride + oxytetracycline hydrochloride (Cu + Ox), gentamicin + oxytetracycline hydrochloride (Gen + Ox), and gentamicin sulphate (GenS). Copper-resistance genes (*copLAB*) were detected by PCR analysis among isolates.

**Results:**

Thirty-seven foliage isolates were identified as *Xanthomonas euvesicatoria* (14%), which were associated with bacterial spot disease in jalapeño pepper. Tested *Xanthomonas* isolates were resistant to Cu-based compounds, but susceptible to Cu + Ox. All isolates were susceptible to Gen + Ox and GenS. *CopLAB* genes were detected in all but one strain.

**Conclusions:**

*X. euvesicatoria* (formally *X. perforans*) may be considered as an emerging pathogen of bacterial spot pepper in Mexico. Among disease management strategies, alternatives to copper should be taken into consideration.

## Introduction

Chili pepper crop (*Capsicum annuum* L.) production in Mexico is an important agricultural activity, because of its high profitability, demand, and consumption. Chihuahua State represents the second most important pepper producer in the country (SIAP, 2018). In Mexico, pepper crop yield reaches up to 25.51 ton/ha. However, diseases such as bacterial spot reduce yield to up to 66%, which represents about $7,500 dollars loss per ha (SIAP, 2018; Sharma & Bhattarai, 2019). Bacterial infection is disseminated among leaves, stems, and fruits, and is characterized by the presence of necrotic spots with chlorotic halos. Spots begin as small watery consistency pustules that become brown with chlorotic halos, resulting in early defoliation, loss of metabolism, and poor product quality (European, Mediterranean Plant Protection Organization, 2013; Keinath, 2019). Potential bacterial spot causative agents include *Xanthomonas euvesicatoria*, *X. gardneri*, *X. euvesicatoria* (formally *X. perforans*), and *X. vesicatoria* (Jones et al., 2004). *X. euvesicatoria*, *X. vesicatoria*, and *X. gardneri* strains have been reported as pathogenic for pepper and tomato, whereas *X. euvesicatoria* (formally *X. perforans*) strains only infect tomato plants (Jones et al., 2004).

After analyzing *Xanthomonas axonopodis* pv. *dieffenbachiae* strains phylogeny against *X. axonopodis* species taxonomy, a reclassification of *X. perforans* and *X. euvesicatoria* as the same *Xanthomonas* species was proposed (Constantin et al., 2016). In fact, authors recommended *X. citri*, *X. phaseoli*, and *X. axonopodis* taxonomic emendations to encompass the PG I, PG III, and PG IV strains, respectively, and *X. perforans* and *X. alfalfae* reclassification as *X. euvesicatoria* to encompass all *X. euvesicatoria* strains as PG II strains. In addition, the reclassification of *X. campestris* pv. *vitians* has been recently emended (Morinière et al., 2020). *X. euvesicatoria* (formerly *X. perforans*) presence in pepper crops has been reported in some studies, which is considered atypical. In fact, strains identified as *X. perforans* were isolated from pepper plants from Florida and Alabama (Potnis et al., 2015; Newberry et al., 2019). Following reclassification changes, the presence of *X. perforans*, now identified as *X. euvesicatoria*, infecting pepper plants, results reasonable. In Mexico, *X. euvesicatoria* and *X. perforans* have been reported infecting tomato, but not pepper plants (Timilsina et al., 2015).

Bacterial spot management includes the use of certified healthy seeds and pathogen-free seedlings, seed treatment with hot water, weed control, and crop rotations (Moura et al., 2020). Furthermore, copper applications or antibiotics are used to control the bacterial spot on tomato and pepper crops (Keinath, 2019). However, antibiotics- or copper-resistant *Xanthomonas* spp. in vegetable crops have been widely distributed in various regions of the globe (Hamza et al., 2010; Abbasi et al., 2015; Areas et al., 2018; Strayer-Scherer et al., 2018; Griffin et al., 2017; Zhang et al., 2013). Bacterial control has been difficult because of its high variability (European, Mediterranean Plant Protection Organization, 2013). Knowledge of the causative agent and antibiotics resistance profile will allow the implementation of more effective control strategies. The presence of copper-resistant or copper-tolerant *Xanthomonas* species causing bacterial spot in Mexico has been reported since 2001 (Carrillo-Fasio et al., 2001). However, along the Chihuahua pepper crop area, their prevalence among infective strains and persistence after copper-based products application for their control are still unknown. The leading evolutionary processes to *X. perforans* new lineages emergence was identified in Alabama, where this species has expanded its host range (it had previously been reported as tomato pathogen, but not infecting pepper) (Newberry et al., 2019). Newberry et al. (2019) also reported a new effector (PthXp1) and different phylogenetic groups that have undergone independent recombination events from multiple *Xanthomonas* species, suggesting a continuous gene flow between Xanthomonadales in association with several host plants, resulting in new lineages emergence. Therefore, the aim of this study was to isolate and identify *Xanthomonas* species infecting plants along the Central South pepper-producing area of Chihuahua, determine their susceptibility to copper-based and oxytetracycline and gentamicin antibacterial compounds, and identify copper-resistance related genes among *Xanthomonas* isolates to discriminate between resistance or tolerance to copper.

## Materials and Methods

General microbiology culture media and reagents for assays and biochemical tests were purchased from Sigma-Aldrich Química de México (Toluca de Lerdo, MX), unless otherwise specified, whereas molecular biology assay reagents were obtained from Promega Corp. (Madison, WI, USA).

### Samples collection

Thirty commercial pepper cultivation lots from the South Central region of Chihuahua State were selected, collecting five samples per lot from plants with bacterial spot symptoms. Samples were then bagged in zip-lock bags, labeled, and transported at 4 °C to our facilities for laboratory processing.

### Causative agent isolation

Leaves with signs of disease were surface sterilized by dipping in 0.5% sodium hypochlorite for 2 min, followed by three rinses in sterile distilled water. Small leaf sections were cut from the spot margin and individually macerated in 500 µL of sterile distilled water, using a sterile mortar and pestle. Resulting suspensions were streaked onto nutrient agar (NA; BD Difco Laboratories, Sparks Maryland, MD, USA) (Shaad, Jones & Chun, 2001) and plates incubated at 27 °C for 48 h, after which they were examined for bacterial colonies development. Pure cultures of bacterial strains were obtained by colony sub-culturing. Collected bacteria cultures were suspended in sterile 0.85% saline solution and stored at 4 °C, until use.

### Phenotypic characterization

Bacterial isolates were subjected to standard biochemical and physiological testing (Shaad, Jones & Chun, 2001). Strains were cultured in semi-selective SX medium (10 g starch, 1 g beef extract, 5 g ammonium chloride, and 2 g K_2_HPO_4_), 1 mL of methyl violet 2B (1% solution in 20% ethanol), 2 mL of methyl green (1% aqueous solution), 15 g agar, and 5 mL of cycloheximide (5.0 g to 10 mL methanol and adjusted to 100 mL with sterile water), and were negative for anaerobic growth and fluorescence development on King’s B medium (20 g proteose peptone, 1.5 g K_2_PO_4,_ 1.5 g MgSO_4_.7H_2_O, 15 mL glycerol, and 15 g agar).

Amylolytic activity of bacterial cultures was tested on nutrient agar plates (BD Difco Laboratories, Sparks Maryland, MD, USA), containing 0.2% soluble starch (Jones et al., 2004). In addition, we performed Gram staining (Hycel^®^, Zapopan, Jalisco, México), oxidase and catalase activities, aerobic/anaerobic growth, and colony characteristics on yeast extract dextrose calcium carbonate agar medium (YDC), containing 10 g/L yeast extract (BD Difco Laboratories, Sparks Maryland, MD, USA), 20 g/L dextrose, 20 g/L calcium carbonate (Fagalab, Sinaloa, México), and 15 g/L agar (BD Difco Laboratories, Sparks Maryland, MD, USA). In YDC medium, *Xanthomonas* species develop yellow, convex, and mucoid colonies. *X. campestris* ATCC 1395 strain was used as a positive control in all tests.

### DNA extraction

DNA extraction was achieved by the phenol-chloroform modified method (Bardakci & Skibinski, 1994). In brief, 1.5 mL of each bacterial isolate grown in nutrient broth medium for 24 h, was centrifuged twice at 12,000 rpm for 5 min at 4 °C, after which supernatant was discarded. Next, 100 μL of 10% sodium dodecyl sulfate, 5 M NaCl, and 10% cetyltrimethylammonium bromide were added, and mixture was homogenized by vortexing at 3,400 rpm (VX-200; Labnet International, Inc., Edison, NJ, USA), followed by incubation for 10 min at 65 °C in a water bath. After this, 750 μL of phenol/chloroform/isoamyl alcohol (25:24:1) mixture was added, mixed by vortexing at 3,400 rpm and centrifuged at 12,000 rpm for 5 min at 4 °C. In order to precipitate the DNA, supernatant was collected, and 500 μL isopropanol were added. The mixture was then carefully stirred by vortexing at low speed 2,000 rpm and placed in the freezer at 20 °C for 24 h, after which solution was centrifuged at 12,000 rpm for 10 min at 4 °C and supernatant was discarded. The precipitate was then washed twice with 300 μL of 70% ethanol, centrifuged for 10 min, dried at 25 °C for 30 min, and suspended in 50 μL of Tris-EDTA (TE) buffer (10 mM Tris-HCl and 1 mM disodium ethylenediaminetetraacetic acid (EDTA) solution at pH 8.

### PCR protocol

Samples concentration was obtained with the Eppendorf BioSpectrometer basic (Eppendorf do Brasil Ltda., Mexico City) and adjusted to 5 ng/μL. To determine the causative agent of spot disease, a multiplex PCR was performed, using the specific primers pairs BsXeF/BsXeR, BsXvF/BsXvR, BsXgF/BsXgR, and BsXpF/BsXpR for *X. euvesicatoria*, *X. vesicatoria, X*. *gardneri*, and *X. perforans*, respectively (Koenraadt et al., 2009) (Table 1) and the following amplification program: an initial denaturation cycle at 94 °C for 5 min, then 35 cycles of denaturation at 94 °C for 30 s, alignment at 64 °C for 1 min, and extension at 72 °C for 1 min, and a final extension cycle at 72 °C for 7 min. Results were analyzed by electrophoresis in a 1.5% agarose gel, using 1X ethidium bromide as an intercalating agent, for 120 min and observing in a GenLogic 200 photodocumenter (Kodak, New York, NY, USA).

**Table 1:** PCR primers used to identify the causative agent of pepper bacterial spot disease^a^. Selected primers used to identify the causative agent of pepper bacterial spot disease

Primer name	5′3′sequence	Amplicon size (bp)	Ann. Temp. (°C)	Target species
Bs-XeF	CATGAAGAACTCGGCGTATCG	173	64	*Xanthomonas euvesicatoria*
Bs-XeR	GTCGGACATAGTGGACACATAC			
Bs-XvF	CCATGTGCCGTTGAAATACTTG	138	64	*Xanthomonas vesicatoria*
Bs-XvR	ACAAGAGATGTTGCTATGATTTGC			
Bs-XgF	TCAGTGCTTAGTTCCTCATTGTC	154	64	*Xanthomonas gardneri*
Bs-XgR	TGACCGATAAAGACTGCGAAAG			
Bs-XpF	GTCGTGTTGATGGAGCGTTC	197	64	*Xanthomonas perforans*
Bs-XpR	GTGCGAGTCAATTATCAGAATGTGG			

**Note:**

a Koenraadt et al. (2009).

#### *copLAB* genes detection

Detection of *cop*LAB genes was performed in the selected isolates from the antimicrobial susceptibility test. Primers used for copper resistance genes were described by Behlau et al. (2013). PCR reaction mixture were performed using a volume of 25 μL. Each PCR reaction mixture consisted of 25 μL sample mixed with 15.5 μL sterile water, 5 μL of 5X PCR buffer, 1.5 μL of 25 mM MgCl_2_, 0.8 μl of 25 mM dNTP’s (EUA; PROMEGA, Madison, WI, USA), 1 μL of each primer (10 μM stock concentration), 1 μL of 5 ng/μL DNA template, and 0.2 μL of 5 U/μL Taq DNA polymerase (PROMEGA, Madison, WI, USA). PCR reactions were performed following the Behlau et al. (2013) protocol. Results were analyzed by electrophoresis in a 2.0% agarose gel, using 1X ethidium bromide as an intercalating agent, for 120 min and observed under a GenLogic 200 photodocumenter (Kodak, New York, NY, USA).

### Pathogenicity test

Bacterial isolates were evaluated for their pathogenicity on pepper seedlings (*Capsicum annuum* var M Southern Star Seeds S. de R.L. de C.V., Mexico City). Seedlings were grown in 10-cm diameter pots with sterile expanded perlite particles, under a 16 h light at 28 °C and 8 h dark at 18 °C. Pathogenicity tests were conducted on 30-d-old pepper seedlings. Before and after testing, seedlings were conditioned two days in darkness, with relative humidity above 90% in a closed 27-gallon plastic chamber (Plastic Trends, Jalisco, Mexico). Bacterial suspensions were prepared from a 24-h-old culture grown on nutrient broth medium at a concentration of 1 × 10^8^ colony forming units (CFU) per milliliter, corresponding to an optical density (OD) of 0.01 at 600 nm by UV-visible spectrophotometry (Evolution 60 S; Thermo Fisher Scientific Inc., Waltham, MA, USA) (Mbega et al., 2012). Next, 10 µL of each bacterial suspension were infiltrated into leaves using Plastipak SFP 1 mL syringes (Becton-Dickinson, Brooklyn, NY, USA), and sterile distilled water as a control. Three leaves were infiltrated with two dots in five seedlings for each bacterial isolate. Disease severity was determined after 5 and 10 d, calculating the leaf damage percentage, using the Canopeo app (https://canopeoapp.com). Canopeo is an application that uses a red, green, and blue (RGB) system. It produces pixels analysis according to R/G, B/G ratios, and the green index excess. Results are interpreted in binary images where white pixels represent the pixels that satisfied the selection criteria (green canopy), whereas black pixels represent the pixels that did not comply with the selection criteria (not green canopy) (Patrignani & Ochsner, 2015). Severity was evaluated considering green percentage present in leaves. A 100% value was associated with healthy leaves, whereas lower percentages were considered as *Xanthomonas* damaged leaves.

### Copper, gentamicin, and oxytetracycline susceptibility testing

Susceptibility of 13 isolates to antibacterial products was evaluated by the broth dilution method (Wiegand, Hilpert & Hancock, 2008).

Tested isolates were selected based on the highest disease severity evidenced in the pathogenicity test, as well as five strains with medium, and other four showing low severity. The evaluated products were Glucob Plus^®^ (8% copper gluconate + 1% citric acid (CuG)), Coboxy^®^ (45% copper oxychloride + 37.7% oxytetracycline hydrochloride (Cu + Ox)), Final Bacter (2% gentamicin + 6% oxytetracycline hydrochloride (Gen + Ox)), gentamicin sulphate (GenS), and copper sulphate pentahydrate (CuS). For minimum inhibitory concentration (MIC) analysis, antimicrobial dilutions were prepared at 4, 8, 16, 32, 64, 128, 256, 512, and 640 µg/mL in tryptone soy broth (TSB). Microbial resistance was defined by *Xanthomonas* growth on copper compounds at 200 µg/mL (Zhang et al., 2013; Roach et al., 2020), on Ox at 25 µg/mL (Farfán, Benítez & Carvajal, 2014), and on gentamicin at 25 µg/mL (Rojas, Peña & Peña-Vera, 2019). In addition, pH values from all the antimicrobial tested and their dilutions were determined.

Bacterial suspensions were adjusted to 0.5 McFarland standard (Becton, Dickinson & Co., Franklin Lakes, NJ, USA) by turbidimetry, which is equivalent to 1 × 10^8^ CFU/mL in TSB. Bacterial suspension was mixed and diluted to 1:100. Next, 50 μL of bacterial suspension plus 50 μL of antimicrobial solution were placed in a microplate, using 100 µL of TSB as a negative control, and 50 µL TSB + 50 µL of each antimicrobial as blank and TSB + bacterial suspension as positive control. Final inoculum contained 5 × 10^5^ CFU/mL. Microplates were then sealed with Parafilm and incubated at 28 °C for 48 h under constant shaking at 225 rpm. After incubation, ODs were read in a Varioskan Flash microplate reader (Thermo Fisher Scientific Inc., Waltham, MA, USA) at 600 nm for MICs determination. Bacterial growth was confirmed by seeding 10 µL of each treatment plus 990 µL of TSB in NA and incubating at 28 °C for 48 h. Tests were performed in triplicate.

### Data analysis

Results from pathogenicity tests were statistically analyzed by the Minitab Statistical Software v.16. Bacterial spot severity results were evaluated by cluster analysis, using the Spearman average linked method. Euclidean distance measure was performed with the InfoStat software (InfoStat version 2009; Grupo InfoStat, Córdoba, Argentina). Phytopathogens were grouped (clusters) into the following categories: (1) isolates that cause high severity, (2) isolates causing medium severity, and (3) isolates causing low severity. From these categories, some representative microorganisms were selected for antimicrobial susceptibility testing.

Antibacterial agents susceptibility data were analyzed by comparing the ODs of the interactions between *Xanthomonas* and antimicrobials at different concentrations, testing concentrations of resistance MIC values for gentamicin, cupper and oxytetracycline, reported by Rojas, Peña & Peña-Vera (2019), Roach et al. (2020) and Farfán, Benítez & Carvajal (2014), respectively.

## Results

### Isolation and identification of the causative agent

In the present study, we isolated 43 *Xanthomonas* spp. strains from chili pepper leaves with characteristic infection of bacterial spots. Colonial morphology on YDC medium showed yellow, mucoid, smooth, circular, and convex colonies with entire margins (Fig. S1). Xanthomonads were gramnegative, and positive for catalase and KOH test. According to biochemical tests, these bacterial isolates belong to the *Xanthomonas* genus. In addition, 37 isolates (87%) showed strong amylolytic activity, associated with *X. euvesicatoria* (formally *X. perforans*) species, whereas 6 isolates (14%) did not possess amylolytic activity and were associated with *X. euvesicatoria* species.

### Molecular characterization of the causative agent

The amplification of 197 bp fragment of 37 isolates with species-specific primers Bs-XpF/Bs-XpR, confirmed them as *X. euvesicatoria* (formally *X. perforans*). In addition, the amplification of 197 bp fragment of six isolates with species-specific primers Bs-XeF/Bs-XeR, confirmed them as *X. euvesicatoria*. No amplification was observed for negative controls and other species-specific primer pairs did not amplify DNA, which confirmed that the isolates do not belong to *X. vesicatoria* or *X. gardneri* (Fig. S2).

### Pathogenicity tests

**Figure 1:**
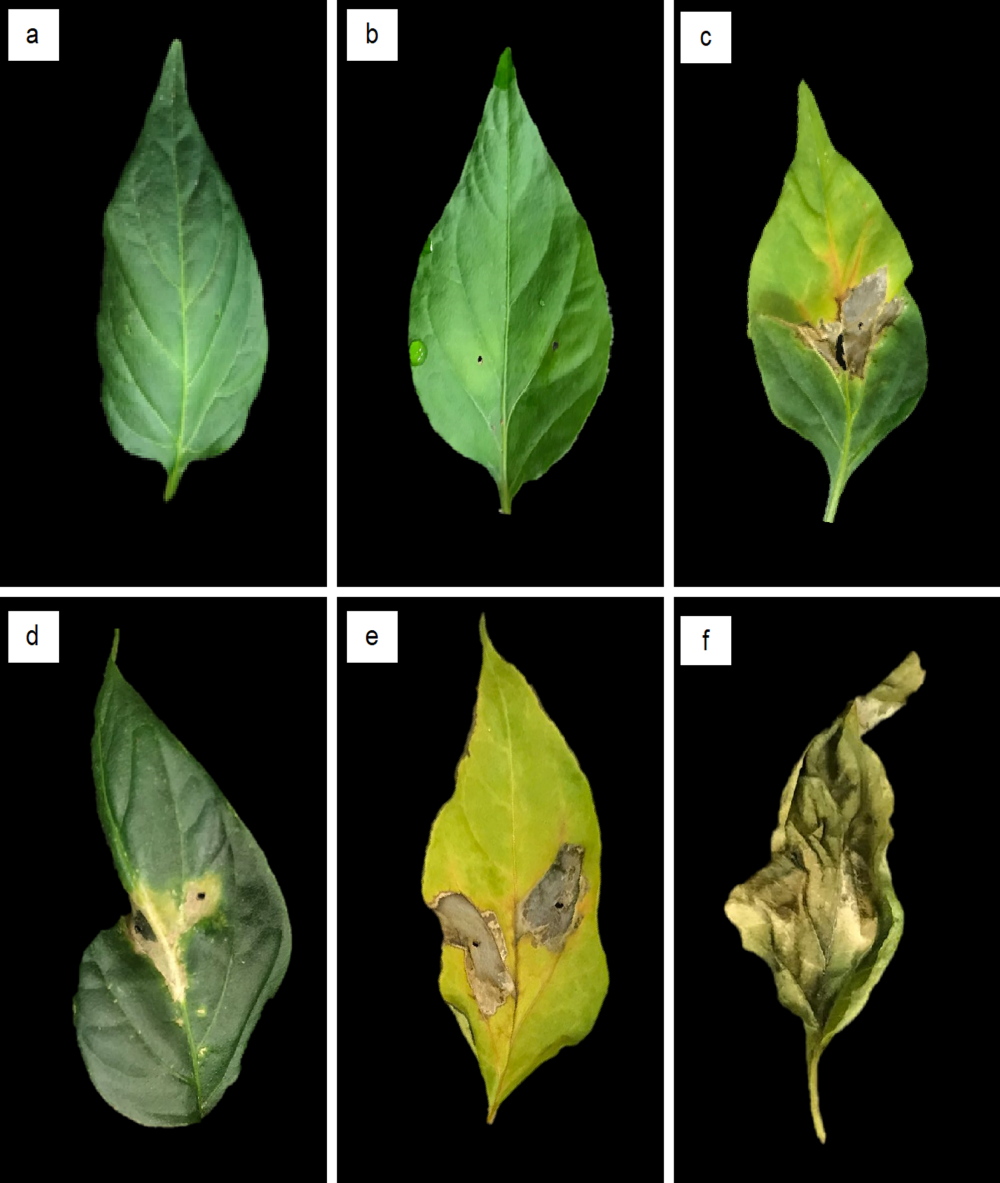
Bacterial spot symptoms induced in jalapeño pepper leaves 10 d after inoculation by *Xanthomonas* isolates from bacterial spot lesions of pepper plants. Bacterial spot symptoms induced in jalapeño pepper leaves 10 d after inoculation by *Xanthomonas* isolates from bacterial spot lesions of pepper plants: (A) healthy leaf treated with 0.25% saline solution; (B) water spots on the upper leaf epidermis; (C) necrotic spot with yellowing and rupture of leaf lamina; (D) necrotic spots with leaf deformation; (E) necrotic spots with leaf chlorosis; and (F) leaf fall and necrosis.

All inoculated pepper leaves with *Xanthomonas* isolates showed signs of bacterial spot. About 5 to 10 d after inoculation, infection was characterized by watery spots, leaves with chlorosis and perforation of leaf lamina, and necrotic spots with chlorosis and defoliation. Negative control plants remained healthy (Fig. 1).

Cluster analysis of the severity of bacteria spot Xanthomonads were classified into three conglomerates, according to their virulence on both evaluations (5 d and 10 d); 9.3% of isolates [all X. *euvesicatoria* (formally *X. perforans*)] caused the highest damage to pepper leaves, from 5 d to the end of the evaluation period, with a final severity of 92.6%. In addition, 25.6% of isolates (ten *X. perforans* and one *X. euvesicatoria*) caused medium damage with 76.8% final severity, and 65.1% (23 isolates were identified as *X. perforans* and 5 as *X. euvesicatoria*) induced low damage with 29.8% severity (Table 2). Medium-severity and low-severity groups showed 54.0% and 13.9% increases in damaged pepper leaves, respectively (Fig. 1).

**Table 2:** Severity of bacterial spot isolates in jalapeño chili pepper seedlings. Indicates the *Xanthomonas* isolates that resulted in higher bacterial spot severity in jalapeño chili pepper seedlings.

Conglomerate	Severity description	Isolate given number	Severity (%)		Isolates^b^
			5 days^a^	10 days	
1	High	4	67.46	92.58	*Xp*21, *Xp*47, *Xp*60, *Xp*69
2	Medium	11	22.72	76.75	*Xp*19, *Xp*24, *Xp*25, *Xp*34, *Xp*43, *Xp*45, *Xp*48, *Xp*50, *Xp*61, *Xe*65, *Xp*66
3	Low	28	15.90	29.83	*Xp*1, *Xp*2, *Xp*3, *Xe*4, *Xp*5, *Xp*6, *Xp*11, *Xp*14, *Xe*15, *Xp*16, *Xp*20, *Xp*22, *Xe*23, *Xp*30, *Xp*31, *Xp*35, *Xp*36, *Xe*37, *Xp*41, *Xe*42, *Xp*46, *Xp*49, *Xp*52, *Xp*53, *Xp*54, *Xp*56, *Xp*58, *Xp*63

**Notes:**

a Days after leaf inoculation.

b *Xanthomonas euvesicatoria* (*Xe*) and *X. perforans* (*Xp*).

### Antimicrobial susceptibility testing

Evaluated *Xanthomonas* strains were resistant to CuS and CuG at all concentrations tested. However, all strains were susceptible to Cu + Ox at ≥ 32 µg/mL. In addition, strains did not grow in Gen + Ox or Gen at 32 µg/mL. Furthermore, microbial growth from *Xp47* and *Xp60* (the most virulent strains) was inhibited by Cu + Ox at 4 µg/mL. In general, 47% of strains did not grow when exposed to Gen at > 4 µg/mL. Since *Xanthomonas* strains were resistant to CuS and CuG, the MIC could not be determined. Calculated MIC of Cu + Ox was from 4 µg/mL to 32 µg/mL, MIC of GenS was from 4 µg/mL to 32 µg/mL, and MIC of Gen + Ox was 32 µg/mL (Table 3). After analyzing the pH values of the antimicrobial agents tested and their dilutions, pH ranged from 6.48 to 7.38 (Table S1).

**Table 3:** Susceptibility of pepper bacterial spot-associated *Xanthomonas* strains^a^ to copper-based compounds, oxytetracycline, and gentamicin. Higher or lower tested concentrations showing the same result as the last one were not depicted.

Compouds	Conc. (μg/mL)	Low				Medium					High				MIC
		*Xe*4	*Xp*16	*Xp*3	*Xp*11	*Xp*25	*Xp*43	*Xp*48	*Xp*50	*Xp*6	*Xp*21	*Xp*47	*Xp*60	*Xp*69	
Copper Sulphate	640	+	+	+	+	+	+	+	+	+	+	+	+	+	ND
Glucob Plus^®^ (8% Copper Gluconate + 1% Citric Acid)	640	+	+	+	+	+	+	+	+	+	+	+	+	+	ND
Coboxy^®^ (45% Copper Oxychloride + 37.7% Oxy-tetracycline Hydrochloride)	4	+	+	+	+	+	+	+	+	+	+	–	–	+	4–32
	8	+	+	+	+	+	+	–	+	+	–	–	–	+	
	16	–	–	+	+	–	+	–	+	+	–	–	–	+	
	32	–	–	–	–	–	–	–	–	–	–	–	–	–	
Final Bacter^®^ (6% Oxy-tretracycline + 2% Gentamicin	16	+	+	+	+	+	+	+	+	+	+	+	+	+	32
	32	–	–	–	–	–	–	–	–	–	–	–	–	–	
Gentamicin	4	–	+	+	–	–	+	–	+	–	+	–	+	+	4–32
	8	–	–	–	–	–	+	–	+	–	+	–	+	+	
	16	–	–	–	–	–	+	–	+	–	+	–	+	+	
	32	–	–	–	–	–	–	–	–	–	–	–	–	–	

**Note:**

a *Xanthomonas euvesicatoria (Xe)* and *X. euvesicatoria* formally *perforans* (*Xp*); no bacterial growth (−); satisfactory bacterial growth on TSB (+); and minimum inhibitory concentration (MIC).

### Copper resistance genes detection

**Figure 2:**
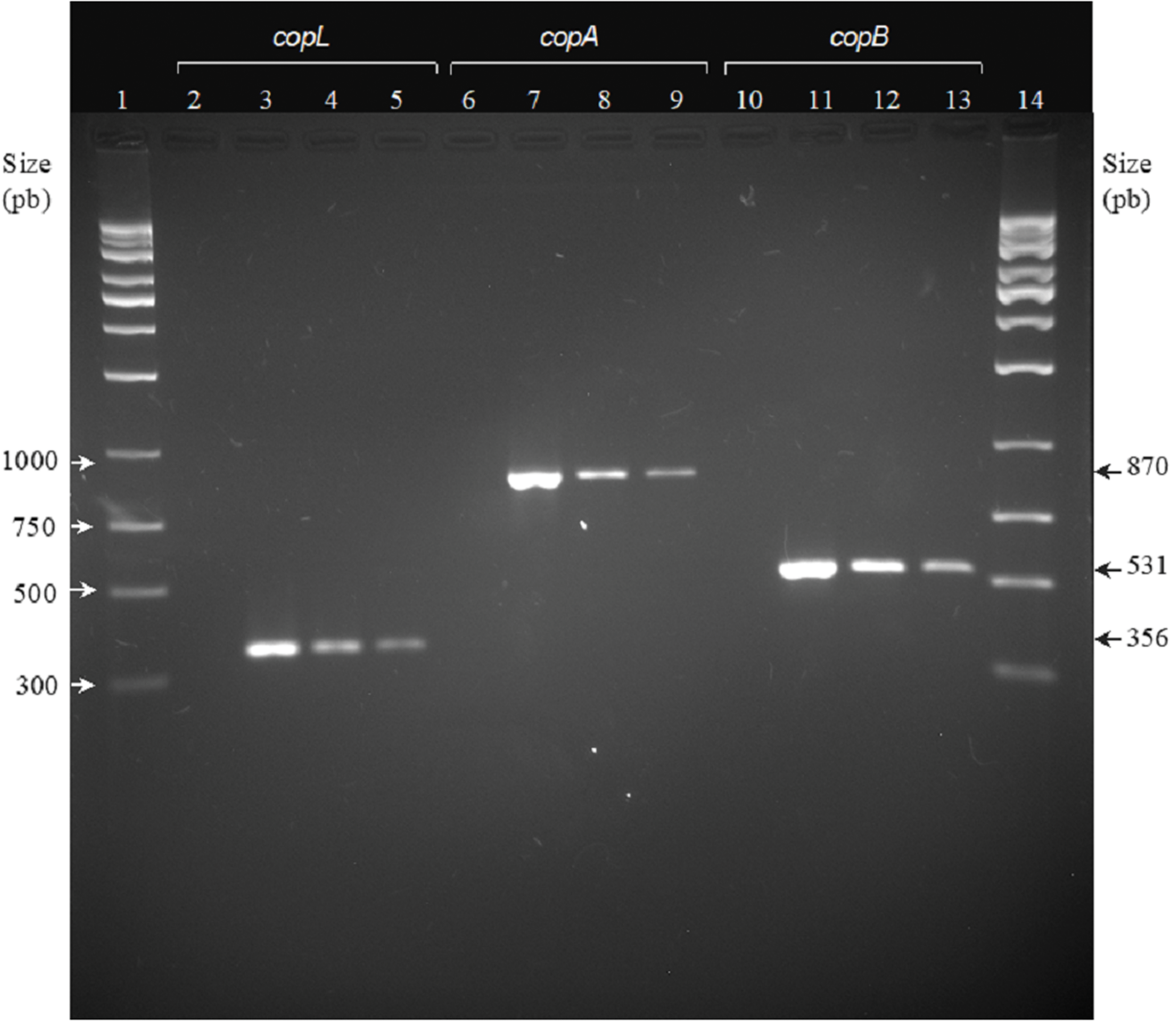
Agarose gel electrophoresis. PCR analysis of copper resistance (CuR) genes *cop*L, *cop*A, and *cop*B. Lanes: (1) 1,000-bp DNA weight ladder; (2,6 and 10) negative control; (3, 7, and 11) *cop*L, *copA*, and *cop*B of *Xanthomonas campestris* ATCC 1395, respectively; (4, 8, and 12) *cop*L, *cop*A. and *cop*B of *X. euvesicatoria* (formally *X. perforans*) Xp48, respectively; (5, 9, and 13) *cop*L, *cop*A. and *cop*B of *X. euvesicatoria* (formally *X. perforans*) Xp60, respectively.

Most *Xhantomonas* strains showed resistance to copper, since *cop*LAB genes were detected. The *cop*L, *cop*A, and *cop*B genes had a molecular weight of 356, 870, and 531 bp, respectively (Fig. 2). However, *cop*LAB genes were not detected in the *Xp*3 strain.

## Discussion

*Xanthomonas euvesicatoria* (formally *X. perforans*) has not been reported as causative agent of pepper bacterial spot in Chihuahua, México. Previous studies have reported *X. euvesicatoria* or *X. campestris* pv. *vesicatoria* (name prior to the reclassification by Jones et al. (2004)) as causative agents of this disease (Guigón-López & González-González, 2001; Chávez-Dozal et al., 2012; Robles-Hernández et al., 2017). Moreover, *X. euvesicatoria* (formally *X. perforans*) strains were recently isolated from pepper plants in USA, suggesting a recent expansion of these species host, implying an emerging threat to pepper production (Potnis et al., 2015; Newberry et al., 2019). This might be related to the continuous flow of genes between *Xanthomonas* related to various plant hosts, which may result in the appearance of new strains of pathogens with the potential to affect other hosts (Newberry et al., 2019). In this study, we only identified *Xanthomonas* species infecting pepper plants and identified them as the bacterial spot causative agents, but evolutionary changes or gene transmission that may led to *X. euvesicatoria* (formally *X. perforans*) attacking the pepper crop were not assessed. Pathogenicity test results demonstrated that all *X. euvesicatoria* strains isolated from pepper plants indeed infected pepper, as previously reported (Jones et al., 2004; Newberry et al., 2019).

Our main objective when analyzing severity by cluster was to group the 43 isolates upon their potential bacterial spot virulence (high, medium, and low), in order to select those from different virulence group to evaluate their susceptibility to antimicrobial agents commonly used in the studied area. This is important from an epidemiological point of view, since pepper is one of the most important crops in Chihuahua, México with a production of 676,463 tons a year (SIAP, 2018). The presence of *X. euvesicatoria* (formally *X. perforans*) in jalapeño pepper plants in South Central Chihuahua, might be due to importation of contaminated seeds, which is a common mechanism for spreading this pathogen (Moura et al., 2020).

In this study, we selected the plate microdilution technique due to its data reproducibility and low cost, thus allowing more antibiotics evaluation with high sensitivity even at low concentrations (Wiegand, Hilpert & Hancock, 2008; Ramírez & Castaño, 2009). Microdilution was performed in three TSB media at pH ranging from 6.48 to 7.38.

In general, as the copper concentration increased, the pH decreased (Franklin et al., 2000). In fact, copper availability in complex culture media such as the one selected in this study, TSB, is affected at low pH values (Hasman et al., 2009). Since the pH values were close to normal (pH = 7), the probability that this physicochemical factor changed the copper availability and the susceptibility results is rather low. Indeed, since all values were close to normal, regardless of the antimicrobial agent and dilutions tested, this may indicate that TBS medium may function as a buffer.

Several studies on copper-resistance by bacterial spot-associated *Xanthomonas* in pepper and tomato have been reported (Carrillo-Fasio et al., 2001; Martin, Hamilton & Kopittke, 2004; Quezado-Duval et al., 2005; Abbasi et al., 2015; Carvalho et al., 2019; Roach et al., 2020; Valarmathi, 2020). In the present study, *Xantomonas* isolates were resistant to copper-based compounds, since all grew at Cu concentrations as high as 640 µg/mL, which is six times higher than that considered as an indicator of copper-resistance (200 µg/mL) (Roach et al., 2020).

Taylor & Reeder (2020) studied the scale and diversity of managing crop recommended antibiotics based on the MICs and the crops and diseases/problems to be selected. They selected low and middle-income countries, grouped by WHO regions, using an international database, comprising over 400,000 records for eight years. Data analysis revealed that antibiotics recommendation and application crops were higher than expected. For instance, within America region (no USA), antibiotics are recommended in 33% of the analyzed countries, where oxytetracycline and gentamicin are mostly used. Out of the recommendations, antibiotic application was in 60% of the diseases/problems where “bacteria” was the causal agent and where in 13% of the cases copper was recommended as well. Out of 10 crops, tomato was the second crop, whose antibiotic application was mostly recommended.

The extent of antibiotic use in crop production relies on that *Xanthomonas* isolates growth inhibition by the combination of copper and oxytetracycline is higher than that of Gen + Ox. This might be due to the synergism between chemical agents, which has been reported in other studies related to copper-resistance of pepper bacterial spot, showing *X*. *euvesicatoria* and *X. campestris* pv. vesicatoria susceptibility to copper compounds (De Aguiar et al., 2003; Areas et al., 2018).

Furthermore, all isolates showed oxytetracycline susceptibility to Gen + Ox and 47% were completely inhibited by Gen. Oxytetracycline resistance by isolates differs with that reported by Farfán, Benítez & Carvajal (2014), who found *Xanthomonas* sp. resistant to Ox at ≤ 25 µg/mL. However, Quezado-Duval et al. (2005) did not detect resistance of pepper bacterial spot-associated X. *euvesicatoria* (formally *X. perforans*) to this compound, which is commonly used to control peach and nectarine bacterial spot disease by *X*. *arboricola* pv. pruni (Sundin & Wang, 2018; Raman et al., 2020).

Resistance to gentamicin at 25 to 100 µg/mL has been reported by *X. campestris* isolates from cabbage and beans (Rojas, Peña & Peña-Vera, 2019), however, none of the tested isolates in the present study showed resistance to gentamicin.

Within *Xanthomonas* strains, detection of *cop*LAB genes indicated copper resistance (Behlau, Gochez & Jones, 2020). Previous studies showed that in culture medium, *cop*LAB genes regulate copper accumulation through copper-binding proteins generation. This is the main resistance mechanism in *Xanthomonas*, where *cop*LAB has been identified as an operon and the regulatory gene is *cop*L, whereas *cop*A and *cop*B genes encode copper-binding proteins (Cooksey, 1994; Voloudakis, Reignier & Cooksey, 2005). In the present study, *Xanthomonas* strains amplified *cop*LAB genes, which would explain their growth in medium containing 640 µg/mL copper. Although the *Xp*3 strain did not present *cop*LAB genes, it was tolerant to copper. Under environmental conditions, bacteria such as *Xanthomonas* may develop tolerance to copper under homeostasis as a result of the constant copper interaction, which is widely distributed in the environment (Brown, Rouch & Lee, 1992; Martínez-Bussenius, Navarro & Jerez, 2017).

## Conclusions

*Xanthomonas euvesicatoria* (formerly *X. perforans*) might be considered as an emerging pathogen of pepper in Mexico, since isolates resulting in the highest bacterial spot severity against jalapeño pepper plants in this study were identified as cupper-resistant isolates.

Among bacterial spot management strategies, alternative options to copper-based compounds must be evaluated in the Chihuahua state of the North region of Mexico. The use of appropriate antibiotic combinations and doses might be effective for the control of phytopathogenic bacteria such as *Xanthomonas*, avoiding bacterial resistance and producing low impact on the environment. Their potential to protect crops with targeted diagnostics and quickly assess to bacterial populations virulence will not only support management strategies, but also application for quarantine purposes.

## Supplemental Information

10.7717/peerj.10913/supp-1 Supplemental Information 1 Colonial morphology of isolates identified as *Xanthomonas perforans* (Xp) and *X. euvesicatoria* (Xe) in yeast extract dextrose calcium carbonate agar YDC medium after 24 h incubation at 28 °C. Xs = unidentified bacteria.

10.7717/peerj.10913/supp-2 Supplemental Information 2 Polymerase chain reaction multiplex amplification obtained with species-specific primer pairs to *Xanthomonas euvesicatoria* BsXeF/BsXeR, *X. vesicatoria* BsXvF/BsXvR, *X. gardneri* BsXgF/BsXgR, *X. euvesicatoria* formally *X. perforans* BsXpF/BsXpR. Lane 1 and 9: Molecular weight marker 50 bp Low Range DNA Ladder. Lane 2: Negative control. Lane 3 to 5: *X. perforans*. Lane 6 to 8 = *X. euvesicatoria*.

10.7717/peerj.10913/supp-3 Supplemental Information 3 pH values from antimicrobial and concentrations tested^1^. ^1^In TSB medium at starting pH of 7.38.

10.7717/peerj.10913/supp-4 Supplemental Information 4 Pathogenicity and susceptibility bioassays data. Pathogenicity test against jalapeño pepper plants and Xanthomonas isolates susceptibility to antimicrobial agents raw data.

## Abbreviations list

CFU: colony forming units
Cu: copper
CuG: copper gluconate + citric acid
CuS: copper sulphate
Cu + Ox: oxytetracycline and copper oxychloride + oxytetracycline hydrochloride
DNA: deoxyribonucleic acid
EDTA: ethylenediaminetetraacetic acid
Gen + Ox: gentamicin + oxytetracycline hydrochloride
GenS: gentamicin sulphate
MIC: minimum inhibitory concentration
NA: nutrient agar
OD: optical density
PCR: polymerase chain reaction
TE: Tris-EDTA
TSB: tryptone soy broth
YDC: yeast extract dextrose calcium carbonate agar medium
Xe: *Xanthomonas euvesicatoria*
Xp: *Xanthmonas perforans*

## Additional Information and Declarations

### Data Availability

The following information was supplied regarding data availability: Raw data are available in the Supplemental Files.
