# Supplementary figures and images for: Prevalence of Xanthomonas euvesicatoria (formally X. perforans) associated with bacterial spot severity in Capsicum annuum crops in South Central Chihuahua, Mexico
Source: PeerJ. 2021 Feb 15;9:e10913. doi: 10.7717/peerj.10913 (PMC7891084; doi:10.7717/peerj.10913)

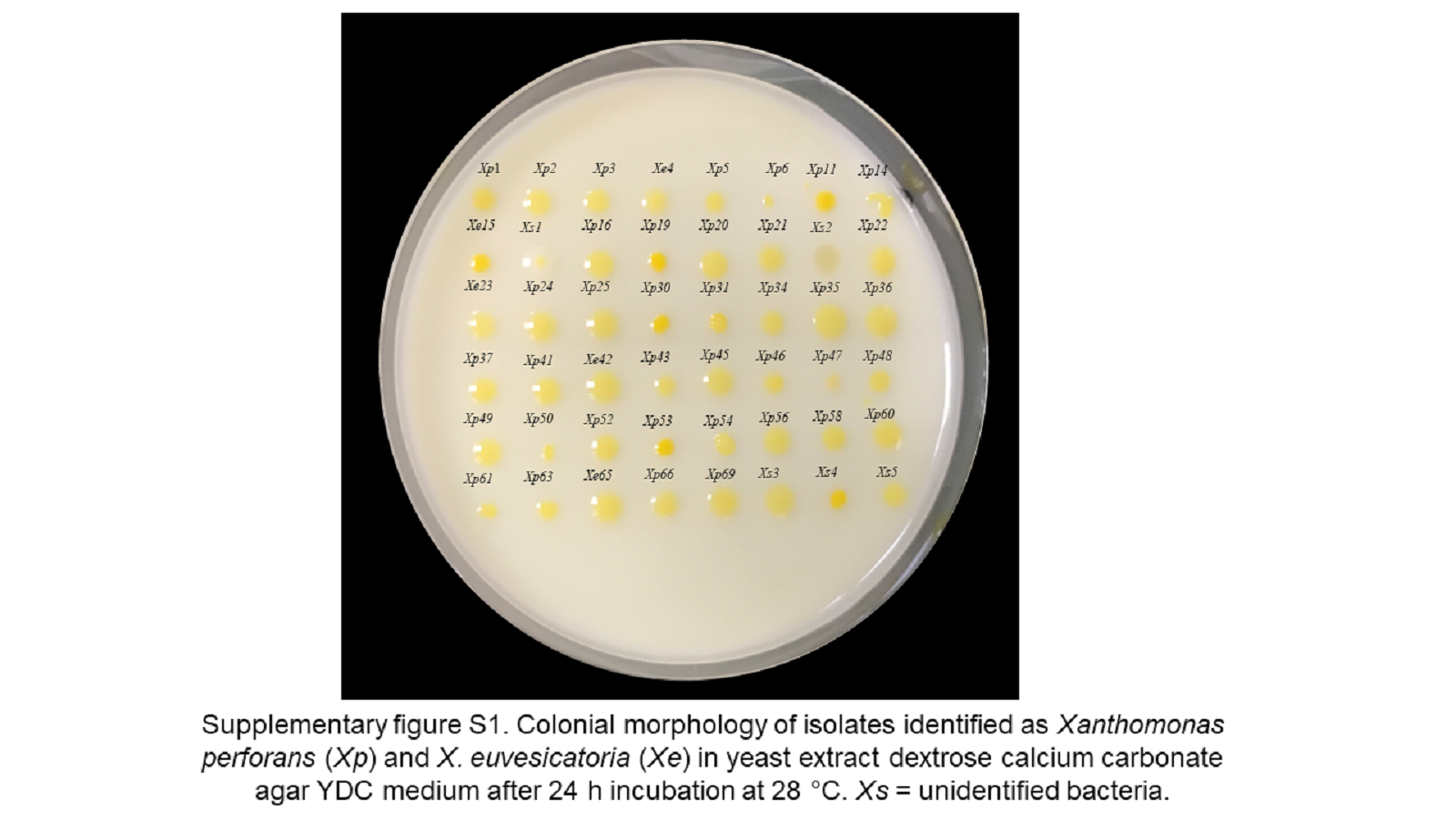

Supplement: Supplemental Information 1 [file peerj-09-10913-s001.png]

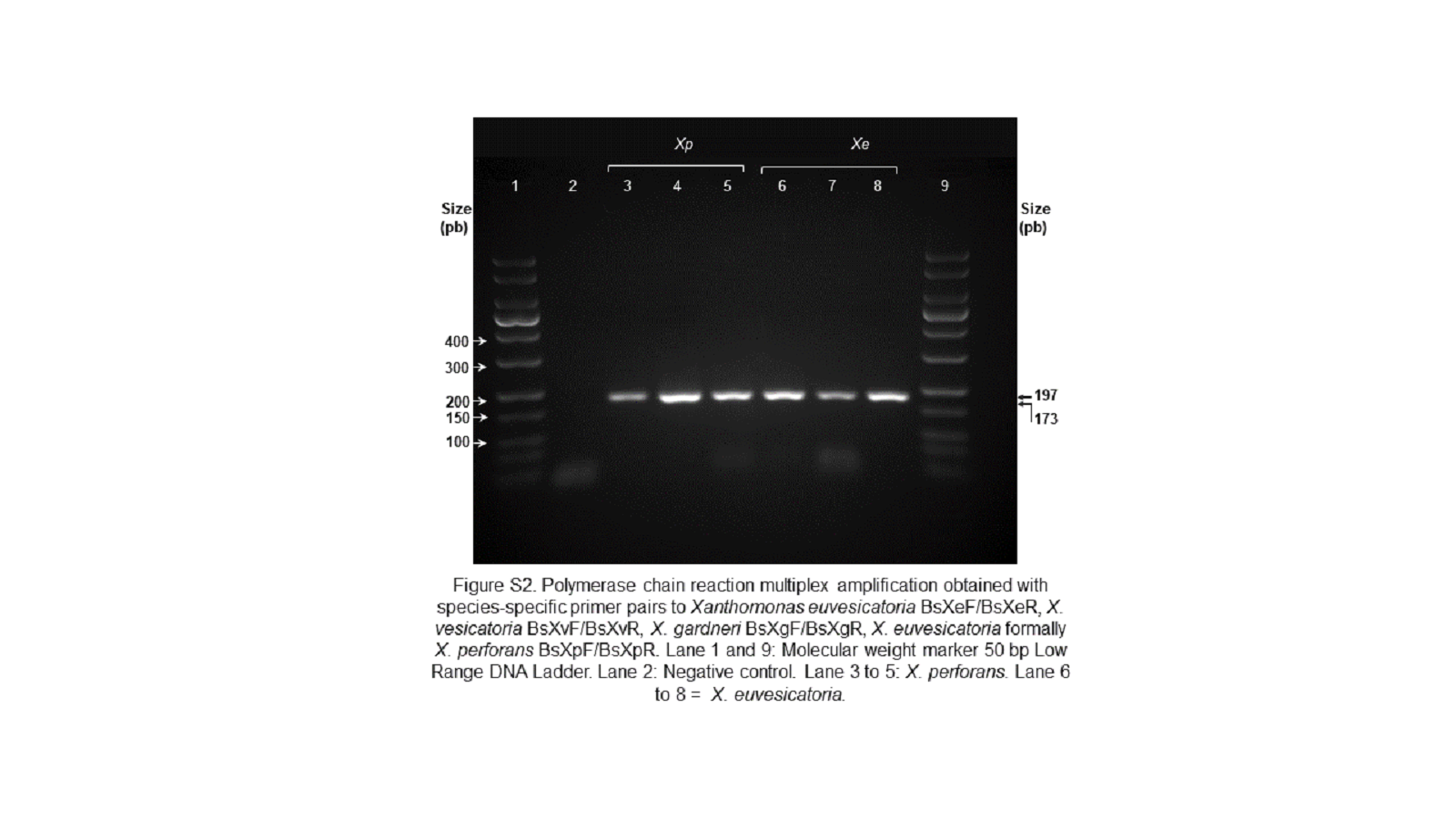

Supplement: Supplemental Information 2 — Lane 1 and 9: Molecular weight marker 50 bp Low Range DNA Ladder. Lane 2: Negative control. Lane 3 to 5: X. perforans. Lane 6 to 8 = X. euvesicatoria. [file peerj-09-10913-s002.png]
