# Supplementary material for: Prevalence of Xanthomonas euvesicatoria (formally X. perforans) associated with bacterial spot severity in Capsicum annuum crops in South Central Chihuahua, Mexico
Source: PeerJ. 2021 Feb 15;9:e10913. doi: 10.7717/peerj.10913 (PMC7891084; doi:10.7717/peerj.10913)
Supplement: Supplemental Information 3 — 1In TSB medium at starting pH of 7.38. [file peerj-09-10913-s003.docx]

| **Table S1. pH values from antimicrobial and concentrations tested^1^** | | | | | |
| --- | --- | --- | --- | --- | --- |
|  | **Antimicrobial agent** | | | | |
| **Concentration (μg/mL)** | **Glucob Plus** | **Copper sulfate pentahydrate** | **Coboxy** | **Final Bacter** | **Gentamicin sulfate** |
| 4 | 7.16 | 7.14 | 7.16 | 7.16 | 7.14 |
| 8 | 7.15 | 7.15 | 7.15 | 7.15 | 7.14 |
| 16 | 7.15 | 7.13 | 7.13 | 7.14 | 7.14 |
| 32 | 7.14 | 7.12 | 7.12 | 7.15 | 7.14 |
| 64 | 7.12 | 7.08 | 7.1 | 7.12 | 7.13 |
| 128 | 7.1 | 7.01 | 7.05 | 7.08 | 7.12 |
| 256 | 7.05 | 6.89 | 6.95 | 7.01 | 7.08 |
| 512 | 6.94 | 6.62 | 6.76 | 6.87 | 7.04 |
| 640 | 6.89 | 6.48 | 6.67 | 6.79 | 7.00 |

^1^In TSB medium at starting pH of 7.38
